# Execution by Electricity

**Published:** 1889-05

**Authors:** 


### EXECUTION BY ELECTRICITY.

The following is from the report of New York Medico-Legal Society, composed of Drs. Frank Peterson, R. Ogden Doremus, Frank H. Ingram, and J. M. Bleyer.

"The electric current, in passing from the top of the head to the small of the back will be diffused throughout a great part of the brain, and all of the tissues of the neck. The medulla oblongata—a part of the brain which is most vital—together with all of the great nerves of the neck and the spinal cord, which exercise jurisdiction over the movements of the lungs and heart, will be thoroughly permeated by the current applied in this way. As the seat of consciousness is in the brain, and particularly in the cortex of the cerebrum, it is clear that this faculty of the mind will suffer at once if the current be sufficiently strong. The electric stream flows from the positive to the negative pole, and there might be some possible advantage in placing the positive pole on the vertex of the head, nearest the centre of consciousness, although death in any case will be instantaneous. After mature deliberation, we recommend that the death-current be administered to the criminal in the following manner :

"A stout table covered with rubber cloth, and having holes along its borders for binding, or a strong chair, should be procured. The prisoner, lying on his back or sitting, should be firmly bound upon this table or in the chair. One electrode should be so inserted into the table, or into the back of the chair, that it will impinge upon the spine between the shoulders. The head should be secured by means of a sort of helmet fastened to the table or back of the chair, and to this helmet the other pole should be so joined as to press firmly with its end upon the top of the head. We think a chair is preferable to a table. The rheophores can be led off to the dynamo through the floor or to another room, and the instrument for closing the circuit can be attached to the wall.

"The electrodes should be of metal, not over one inch in diameter, somewhat ovoidal in shape, and covered with a thick layer of sponge or chamois-skin. The poles and the skin and hair at the points of contact should be thoroughly wet with warm water. The hair should be cut short.

"A dynamo generating an electromotive force of at least three thousand volts should be employed. Either a continuous or alternating current may be used, but preferably the latter. The current should be allowed to pass for thirty seconds."

The law provides that execution by electricity shall take place only in Sing Sing, Auburn and Clinton. New York city murderers will be executed at Sing Sing.—*Medical Record.*

